# From Resistance to Relief: IV Levothyroxine in Refractory Hypothyroidism Management

**DOI:** 10.1002/ccr3.71438

**Published:** 2025-11-09

**Authors:** Mohammad Amro, Elias Edward Lahham, Ahmed Jalal Sawafta, Motaz Saifi, Nooreddin Saifi, Fadi Atrash, Salem Billan

**Affiliations:** ^1^ Faculty of Medicine An Najah National University Nablus Palestine; ^2^ Department of Radiation Oncology Augusta Victoria Hospital East Jerusalem Palestine; ^3^ Head and Neck Unit, Joseph Fishman Oncology Center Rambam Health Care Campus Haifa Israel

**Keywords:** hypothyroidism, intravenous, levothyroxine, pseudo‐malabsorptive hypothyroidism, refractory hypothyroidism

## Abstract

This case report details the clinical scenario of a 27‐year‐old female patient diagnosed with idiopathic refractory hypothyroidism. Despite adherence to prescribed oral levothyroxine therapy, she continued to experience persistent symptoms such as fatigue and cold intolerance. The exploration of her condition delved into the complexities of refractory hypothyroidism, encompassing both its diagnostic challenges and therapeutic management. A critical distinction was made between true levothyroxine malabsorption and pseudo‐malabsorption, the latter being attributed to issues with medication adherence. The scarcity of research on intravenous levothyroxine use in such cases was noted, highlighting the novelty of this approach. The patient's favorable response to intravenous levothyroxine administration, marked by complete symptom resolution and sustained euthyroid state over four years, provides valuable insights. This case contributes significantly to the existing knowledge on the idiopathic etiologies of refractory hypothyroidism and advocates for the consideration of intravenous levothyroxine as an effective alternative when oral administration proves inadequate.

**Summary**

- Patients with idiopathic refractory hypothyroidism who have been identified with oral levothyroxine resistance may benefit from IV levothyroxine as it can alleviate their symptoms and assist physicians in differentiating between true and pseudo‐malabsorptive hypothyroidism after excluding any other potential causes.

## 1 Introduction

To restore normal thyroid hormone levels in hypothyroidism, oral levothyroxine sodium (LT4) continues to be the cornerstone of treatment. Levothyroxine absorption can be affected by age, patient compliance, fasting, and the consumption of certain foods or medications [1, 2]. Refractory hypothyroidism can have numerous causes, including poor or nonadherence to levothyroxine daily doses, deiodinase deficiency, and various malabsorption disorders. While weekly oral, suppository, or intramuscular levothyroxine (LT4) injection has been shown in prior research to be a successful treatment for refractory hypothyroidism. However, limited information is currently available on treatment involving the weekly intravenous administration of LT4 in non‐acute settings [3]. Here we present a case of a 27‐year‐old female with idiopathic refractory hypothyroidism who failed to respond to oral levothyroxine, but achieved biochemical and clinical euthyroid with weekly IV LT4. This case highlights the potential role of IV levothyroxine as an alternative in carefully selected patients who remain unresponsive to conventional therapy.

## 2 Case History, Examination, and Investigation

A 27‐year‐old female with a history of papillary thyroid carcinoma has presented with general fatigue and cold intolerance. The patient was diagnosed with papillary thyroid carcinoma at the age of nine and underwent total thyroidectomy. Since then, the patient has been committed to oral levothyroxine (LT4) daily. However, at the age of 22, a recurrence was discovered in the lymph nodes and she had a neck dissection; the pathology was positive for metastasis in 6 out of 20 lymph nodes on the left side and 8 out of 18 lymph nodes on the right side, followed by radio‐iodine ablation (RIA). The patient had another neck dissection at the age of 24, due to disease recurrence. By that time, positron emission tomography (PET) scans showed uptake in the thyroid bed and posterior neck, in addition to increased levels of thyroglobulin, and later in the same year, a biopsy demonstrated papillary thyroid carcinoma in the right lobe. However, the multidisciplinary team decided there was no need for surgery, and the patient will continue to have regular follow‐up visits, PET scans, and daily oral LT4 400 mg.

At the age of 27, and despite being committed to LT4, the patient starts feeling fatigued and shows cold intolerance. Other physical exam findings were normal. Laboratory values showed TSH levels of 66 mIU/L and a Baseline Free T3 was 1.3, Free T4 1.2, hemoglobin of 8 g/dL, and deficiency in vitamin D and B12 where her 25‐oh vitamin D levels were 4.31 ng/mL and her B12 levels were 112 pg/mL. The patient was admitted for further follow‐up, given IV levothyroxine 300 μg daily for four days where the dose was calculated according to the patient's weight at that time 55 kg, and put on IV thyroxine once a month in addition to the oral dose. The patient's status improved after the IV administration, and TSH dropped to 3 mIU/L, The route of administering the medication was determined to be IV since it provides rapid onset of action, complete bioavailability, and more predictable pharmacokinetics than either intramuscular (IM) or subcutaneous (SC) routes, making it particularly suitable for cases requiring swift biochemical correction and symptomatic relief, Recent reviews and case series [3, 4] emphasize that IV therapy remains the preferred route in patients with suspected malabsorption, myxedema, or refractory hypothyroidism unresponsive to oral formulations, particularly in the outpatient setting when managed carefully.

## 3 Differential Diagnosis

Two months later, the patient presented with the same symptoms and required another hospital admission for IV thyroxine due to a suspicion of decreased oral levothyroxine absorption. A duodenal biopsy was taken, which has shown no evidence of *H. pylori* , celiac disease, or any form of dysplasia or malignancy. During that time, the patient had not taken any over‐the‐counter medications and confirmed compliance with the prescribed medications and the patient's adherence to thyroxine was verified through direct questioning, pill counts, and supervised administration. She was instructed to take the medication on an empty stomach, separate from food and supplements, which she consistently followed. No concomitant medications or gastrointestinal conditions that could impair absorption were identified. Despite these measures, TSH remained persistently elevated. This may reflect individual variability in absorption or an unusually high thyroxine requirement, which has been described in the literature, even in the absence of malabsorption syndromes or drug interference.

A comprehensive investigation was conducted to rule out possible causes of refractory hypothyroidism and malabsorption. The patient had no history of gastric bypass, inflammatory bowel disease, or autoimmune conditions such as celiac disease. Furthermore a duodenal biopsy was taken, which has shown no evidence of *H. pylori* , celiac disease, or any form of dysplasia or malignancy. Laboratory evaluation, including serum vitamin levels and anti‐transglutaminase antibodies, showed no abnormalities. No interfering medications, including calcium, iron supplements, cholestyramine, or proton pump inhibitors, were discovered, and no drug–drug interactions were suspected. Detailed pill count records and therapeutic observation were used to verify her adherence, These findings collectively support the exclusion of known pathological or behavioral causes, and the diagnosis of idiopathic refractory hypothyroidism was made by exclusion.

## 4 Outcome and Follow‐Up

**FIGURE 1:**
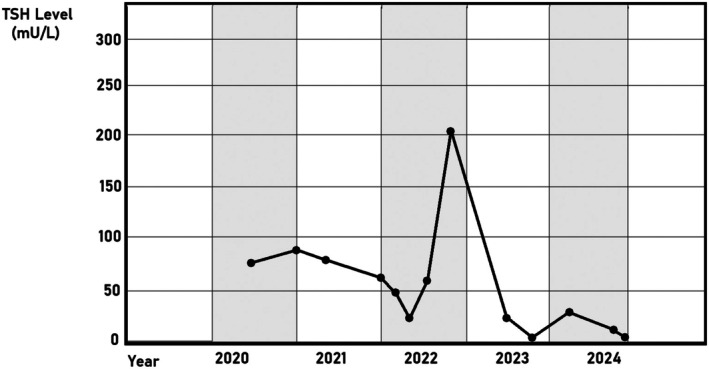
TSH levels peak in late 2022, followed by a rapid decline. After some fluctuations, the levels eventually stabilized by the end of the observed period in 2024.

**FIGURE 2:**
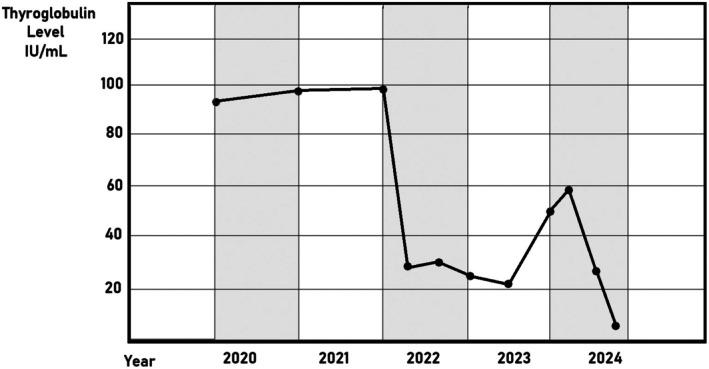
Thyroglobulin levels fall sharply following an early peak in 2022. Despite a slight increase at one point, levels continue to fall and stabilize near the end of the observation period in 2024.

Based on the above‐mentioned findings, and after excluding any other potential causes, the patient was diagnosed with refractory hypothyroidism and was prescribed IV thyroxine 300 μg three times weekly for the last four years, where the dose was selected based on the patient's high oral dose requirement (400 μg daily) and her clinical response, Using the IV‐to‐oral conversion ratio (approximately 75% of oral dose), this approach is consistent with dosing strategies reported in refractory hypothyroidism literature [3]. During the last follow‐up visit, the patient was asymptomatic, showing no abnormalities, with a normal range of TSH (Figure 1), T3, T4, and thyroglobulin (Figure 2). While formal quality‐of‐life questionnaires were not administered, the patient reported significant improvement in energy levels, mood, and daily functioning following the initiation of IV therapy. We acknowledge the value of standardized tools such as the ThyPRO questionnaire and will note this as a limitation and recommendation for future reports. Additionally, the patient is not complaining of any other medical conditions, and was monitored for any complications related to using IV therapy during the four years of follow‐up, and no complications were mentioned.

During the initial evaluation, vitamin D and vitamin B12 deficits were discovered in the patient. In order to prevent possible interference with absorption, she was specifically instructed to take the supplements at a separate time of day than her levothyroxine dosage. She was supplied with the proper supplements for both. Subsequent laboratory tests verified that the levels of vitamin B12 and D had returned to normal. The patient showed no signs of deficiency‐related symptoms during follow‐up, indicating that the correction was successful.

## 5 Discussion

Refractory hypothyroidism, a condition where the thyroid gland does not respond adequately to treatment, can arise from a multitude of factors. Non‐pathological reasons include patient noncompliance, often due to poor adherence to medication schedules, leading to reduced treatment absorption.

Certain medications, such as proton‐pump inhibitors (PPI), histamine receptor blockers, and cholestyramine, along with conditions like irritable bowel syndrome, lactose intolerance, and gastroesophageal reflux disease, can interfere with gastrointestinal absorption. Pregnancy also necessitates specific adjustments in medication dosage across different trimesters. Pathologically, gastrointestinal disorders like inflammatory bowel disease, *H. pylori* infection, and the effects of gastric bypass surgery can contribute to the condition. Furthermore, individual factors such as body mass, gender, and genetic variations specifically in deiodinase D2 can influence the effectiveness of levothyroxine therapy. It is essential to differentiate true malabsorption from pseudo‐malabsorption, which is linked to poor treatment adherence. Idiopathic causes, where the reason remains unknown, account for 10%–20% of cases. Understanding these diverse factors is crucial for the effective management of refractory hypothyroidism [5, 6].

The management of refractory hypothyroidism requires a meticulous and structured approach. Confirming patient adherence to prescribed treatment and proper medication administration techniques is crucial. A thorough review of the patient's medication history is essential to rule out drug interactions that may affect thyroxine levels. Subsequently, conducting tests to investigate potential thyroxine malabsorption is advised, which may include assessing gastrointestinal function and health. For women of childbearing age, a pregnancy test is imperative as pregnancy can alter thyroid hormone levels. Finally, it takes a mix of behavioral, laboratory, and clinical evaluations to distinguish between actual malabsorption and pseudo‐malabsorption. Elevated fecal fat (> 7 g/day), abnormal D‐xylose tests, and nutrient deficiencies despite adequate intake are objective indicators of true malabsorption, which is caused by organic gastrointestinal dysfunction such as Crohn's disease, pancreatic insufficiency, celiac disease (villous atrophy), or following sleeve gastrectomy operation [7, 8]. The absence of physiological malabsorption, on the other hand, is known as pseudo‐malabsorption, which is frequently brought on by pharmaceutical nonadherence, factitious diseases, or inadvertent dietary noncompliance [7]. Directly observed therapy and serum drug monitoring where measurement of free T4 at baseline, 60, and 120 min. Values below a 54% increment of T4 at 120 min are compatible with gastrointestinal LT4 malabsorption, whereas values similar to or above suggest poor compliance. These methods help with ruling out pseudo‐malabsorption [7]. In conclusion, a thyroxine absorption test should be performed to distinguish between pseudo‐malabsorption where the issue is not actual malabsorption but perhaps noncompliance or interference, and true levothyroxine (LT4) malabsorption [6].

Treatment in cases of refractory hypothyroidism is achieved by different methods as proposed in different studies of soft gel and liquid LT4 showing potential in specific groups such as pediatric patients, bariatric surgery patients, patients on PPI, and patients unwilling to delay breakfast [9]. Parenteral administration is another suggested treatment. This includes SC, IM, and IV routes. Hays' mathematical model [10, 11] is used to figure out the doses, and the rates of absorption are twice as high with intramuscular and intravenous administration compared to SC administration. Moreover, intramuscular administration was reported in multiple cases and favored due to the ability to safely adjust the dose once or twice weekly, which proved to be an acceptable alternative in cases of resistant hypothyroidism to achieve a euthymic state in reported patients [11, 12, 13].

Only a limited number of studies in the outpatient setting reported the IV administration of LT4. This was primarily due to its use in hospitalized patients and patients with myxedema. However, given that the IV dose is 75% of the enteral dose and can be administered weekly, it serves as a great substitute for the oral form as evidenced in our study to achieve a euthymic state. The efficacy of non‐oral methods, such as intramuscular and subcutaneous injections, was also noted by Nakano et al. (2019) in patients who had gastrointestinal or compliance‐related treatment difficulties. This method effectively addresses pseudo‐malabsorption and overcomes the factors associated with levothyroxine malabsorption in the GI tract [3, 4, 14, 15].

Ritter et al. [16] reported a case of selective malabsorption in which a patient required intravenous levothyroxine to achieve hormonal normalization, after extensive investigations excluded structural or functional gastrointestinal disease. Similarly, Nagaoka et al. [15] described a patient who required long‐term parenteral LT4 after failing to respond to multiple oral formulations and dosages, highlighting the clinical utility of IV therapy in refractory cases. Although IV levothyroxine therapy is reported in many cases, its broad use is first constrained by cost and accessibility, as IV LT4 is generally only available in hospital settings and is substantially more costly than oral versions. Second, there are real‐world challenges since venous access is necessary where it could increase the catheter infection risk and the lack of data that studies the effects and complications of using IV therapy highlights the necessity of individualized treatment plans and further studies to establish standardized protocols. In our case, these concerns were mitigated through coordinated care, patient education, and regular outpatient follow‐up. The therapy was feasible due to the patient's proximity to a medical facility and strong motivation to maintain euthyroid status. We acknowledge that IV therapy may not be a universal solution and should be considered on a case‐by‐case basis, weighing the risks, benefits, and healthcare resources.

## Author Contributions

**Mohammad Amro:** resources, writing – original draft, writing – review and editing. **Elias Edward Lahham:** conceptualization, resources, supervision, writing – original draft, writing – review and editing. **Ahmed Jalal Sawafta:** writing – original draft, writing – review and editing. **Motaz Saifi:** writing – original draft, writing – review and editing. **Nooreddin Saifi:** writing – original draft, writing – review and editing. **Fadi Atrash:** writing – original draft, writing – review and editing. **Salem Billan:** writing – original draft, writing – review and editing.

## Ethics Statement

Our institution does not require ethical approval for reporting individual cases or case series.

## Consent

Written informed consent was obtained from the patient herself for her anonymized information to be published in this article.

## Conflicts of Interest

The authors declare no conflicts of interest.

## Data Availability

The data used to support the findings of this study are included within the article.

## References

[1] O. Elbasan and D. G. Yavuz , “Refractory Hypothyroidism to Levothyroxine Treatment: Five Cases of Pseudomalabsorption,” Acta Endocrinologica 16 (2020): 339–345. 33363657 10.4183/aeb.2020.339 PMC7748222

[2] G. Ianiro , F. Mangiola , T. A. Di Rienzo , et al., “Levothyroxine Absorption in Health and Disease, and New Therapeutic Perspectives,” European Review for Medical and Pharmacological Sciences 18, no. 4 (2014): 451–456. 24610609

[3] Y. Nakano , K. Hashimoto , N. Ohkiba , et al., “A Case of Refractory Hypothyroidism due to Poor Compliance Treated With the Weekly Intravenous and Oral Levothyroxine Administration,” Case Reports in Endocrinology 2019 (2019): 5986014. 30867970 10.1155/2019/5986014 PMC6379869

[4] H. Liu , W. Li , W. Zhang , S. Sun , and C. Chen , “Levothyroxine: Conventional and Novel Drug Delivery Formulations,” Endocrine Reviews 44 (2023): 393–416. 36412275 10.1210/endrev/bnac030 PMC10166268

[5] P. Fallahi , S. M. Ferrari , and A. Antonelli , “Oral l‐Thyroxine Liquid Versus Tablet in Patients With Hypothyroidism Without Malabsorption: A Prospective Study,” Endocrine 52, no. 3 (2016): 597–601. 26721663 10.1007/s12020-015-0836-y

[6] M. Centanni , S. Benvenga , and I. Sachmechi , “Diagnosis and Management of Treatment‐Refractory Hypothyroidism: An Expert Consensus Report,” Journal of Endocrinological Investigation 40 (2017): 1289–1301. 28695483 10.1007/s40618-017-0706-y PMC5680379

[7] E. Gruneisen , J. W. Yang , and M. R. Pasqua , “Levothyroxine Malabsorption Following Sleeve Gastrectomy,” Endocrinology, Diabetes & Metabolism Case Reports 2025, no. 1 (2025): e240115, https://pmc.ncbi.nlm.nih.gov/articles/PMC11811825/. 10.1530/EDM-24-0115 PMC11811825 39868565

[8] G. M. Rdzak , L. M. Whitman , and S. E. Inzucchi , “Levothyroxine Pseudo‐Malabsorption: Testing and Treatment in the Outpatient Setting,” Therapeutic Advances in Endocrinology and Metabolism 9, no. 7 (2018): 217–222, https://pubmed.ncbi.nlm.nih.gov/29977500/. 29977500 10.1177/2042018818771433 PMC6022974

[9] V. Srinivas and S. O. Oyibo , “Levothyroxine Pseudomalabsortion and Thyroxine Absorption Testing With Use of High‐Dose Levothyroxine: Case Report and Discussion,” Endocrine Practice 16, no. 6 (2010): 1012–1015. 21041167 10.4158/EP10224.CR

[10] C. Virili , P. Trimboli , F. Romanelli , and M. Centanni , “Liquid and Softgel Levothyroxine Use in Clinical Practice: State of the Art,” Endocrine 54 (2016): 3–14. 27473098 10.1007/s12020-016-1035-1

[11] M. T. Hays , “Parenteral Thyroxine Administration,” Thyroid 17 (2007): 127–129. 17316114 10.1089/thy.2006.0283

[12] M. D. L. Á. Garayalde Gamboa , M. Saban , and M. I. Curriá , “Treatment With Intramuscular Levothyroxine in Refractory Hypothyroidism,” European Thyroid Journal 8, no. 6 (2019): 319–323. 31934558 10.1159/000503324 PMC6944946

[13] P. N. Taylor , A. Tabasum , G. Sanki , et al., “Weekly Intramuscular Injection of Levothyroxine Following Myxoedema: A Practical Solution to an Old Crisis,” Case Reports in Endocrinology 2015 (2015): 169194. 26618010 10.1155/2015/169194 PMC4651700

[14] A. Tönjes , S. Karger , C. A. Koch , et al., “Impaired Enteral Levothyroxine Absorption in Hypothyroidism Refractory to Oral Therapy After Thyroid Ablation for Papillary Thyroid Cancer: Case Report and Kinetic Studies,” Thyroid 16, no. 10 (2006): 1047–1051. 17042692 10.1089/thy.2006.16.1047

[15] T. Nagaoka , H. Miyakoshi , T. Takamura , et al., “A Case of Refractory Hypothyroidism Requiring Daily Intravenous Thyroxine,” Journal of International Medical Research 30, no. 4 (2002): 463–465. 12235934 10.1177/147323000203000418

[16] M. J. Ritter , S. Gupta , and J. V. Hennessey , “Alternative Routes of Levothyroxine Administration for Hypothyroidism,” Current Opinion in Endocrinology, Diabetes, and Obesity 27, no. 5 (2020): 318–322, https://pubmed.ncbi.nlm.nih.gov/32740045/. 32740045 10.1097/MED.0000000000000558

